# 2,7(3,6)-Diaryl(arylamino)-substituted Carbazoles as Components of OLEDs: A Review of the Last Decade

**DOI:** 10.3390/ma14226754

**Published:** 2021-11-09

**Authors:** Gintare Krucaite, Saulius Grigalevicius

**Affiliations:** Department of Polymers Chemistry and Technology, Kaunas University of Technology, Radvilenu Plentas 19, LT50254 Kaunas, Lithuania; gintare.krucaite@ktu.lt

**Keywords:** substituted carbazole, amorphous material, charge transporting layer, emitter, host material, organic light emitting diode

## Abstract

Organic light emitting diode (OLED) is a new, promising technology in the field of lighting and display applications due to the advantages offered by its organic electroactive derivatives over inorganic materials. OLEDs have prompted a great deal of investigations within academia as well as in industry because of their potential applications. The electroactive layers of OLEDs can be fabricated from low molecular weight derivatives by vapor deposition or from polymers by spin coating from their solution. Among the low-molar-mass compounds under investigation in this field, carbazole-based materials have been studied at length for their useful chemical and electronic characteristics. The carbazole is an electron-rich heterocyclic compound, whose structure can be easily modified by rather simple reactions in order to obtain 2,7(3,6)-diaryl(arylamino)-substituted carbazoles. The substituted derivatives are widely used for the formation of OLEDs due to their good charge carrier injection and transfer characteristics, electroluminescence, thermally activated delayed fluorescence, improved thermal and morphological stability as well as their thin film forming characteristics. On the other hand, relatively high triplet energies of some substituted carbazole-based compounds make them useful components as host materials even for wide bandgap triplet emitters. The present review focuses on 2,7(3,6)-diaryl(arylamino)-substituted carbazoles, which were described in the last decade and were applied as charge-transporting layers, fluorescent and phosphorescent emitters as well as host materials for OLED devices.

## 1. Introduction

Since the first discovery of organic electroluminescent compounds, huge attention has been devoted to the creation of new materials and optimized multilayer device architectures for viable and practical organic light emitting diodes (OLEDs), which would demonstrate low-driving voltage, high brightness, full-color emission, a long lifetime, and the easy formation of flexible thin-film devices. The commercial benefit of OLED devices is seen in the emergence of digital cameras, mobile cell phones as well as display technologies; however, there is still a strong demand to considerably improve the performance and lifetime of the devices for lighting technologies [1,2,3,4,5].

Formerly, structurally and chemically well-defined, carbazole-based compounds and their characteristics were investigated due to their properties of biological activity [6,7,8]. However, in the last three decades, the carbazole rings or substituted carbazole-containing compounds have been widely investigated in the field of organic optoelectronics, particularly in OLED technologies [9,10,11,12]. The substituted carbazole-based derivatives are widely used as components of the devices due to their good charge injection and transport characteristics in the layers of the materials [13], electroluminescence [14], thermally activated delayed fluorescence [15], improved thermal and morphological stability as well as their homogenous thin film formation properties [16]. In addition, the rather high triplet energies of some aryl(arylamino)-substituted derivatives make them useful components, acting as host materials for the phosphorescent triplet emitters in electrophosphorescent OLED technologies [17].

The 9H-carbazole core as electron donor (D) can be easily substituted at the 9,2,7(3,6)-positions by using different aromatic fragments. Donor–acceptor (D-A) derivatives (9-arylcarbazoles), asymmetric 9,2(3)-diarylcarbazoles as well as symmetric 2,7(3,6)-diarylcarbazoles could be synthesized by using various arylation or amination reactions. D-D fragments containing derivatives are usually used as hole-transporting layer materials or fluorescent emitters [18,19,20]. The D-A and A-D-A structures that have carbazoles are more suitable as host materials for phosphorescent triplet emitters, thermally activated delayed fluorescence (TADF)-based emitters as well as in other less investigated fields such as exciplex components, intramolecular hosts, dopants, etc. [21]. D-A structure derivatives and asymmetric 9,2(3)-diaryl(diarylamino)carbazoles are rather widely described in earlier review articles [22,23]. In the present review, we analyze and present only symmetric 2,7(3,6)-diaryl(arylamino)-substituted carbazoles, which were developed and described in last decade and were applied as charge-transporting layers, fluorescent and phosphorescent emitters as well as host materials for the OLED devices.

## 2. Synthesis of 2,7(3,6)-Diiodo(dibromo)carbazoles and 2,7(3,6)-Diaryl(diarylamino)-substituted Objective Carbazoles

**Scheme 1:**
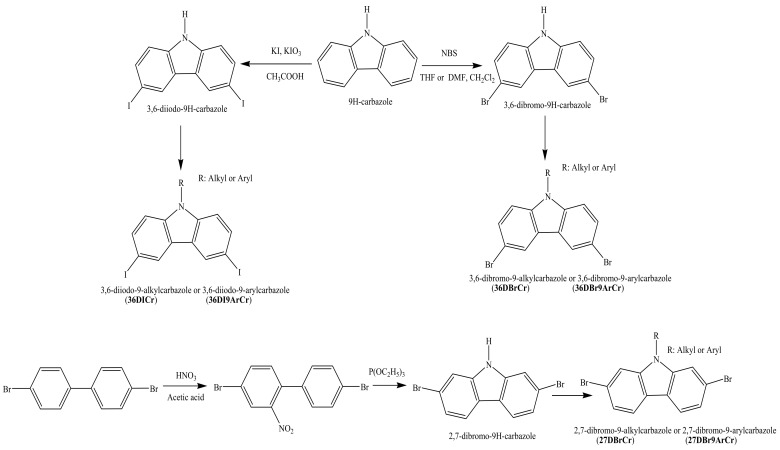
Synthesis of starting compounds: 3,6-diiodo(dibromo)-9-alkyl(aryl)carbazoles and 2,7-dibromo-9-alkyl(aryl)carbazoles.

Both the 3,6-Diiodo-9H-carbazoles and 3,6(2,7)-dibromo-9H-carbazoles are intermediate materials for the preparation of objective carbazole-based derivatives for OLEDs. Structures of the halogenated derivatives are shown in Scheme 1. The bromination reaction of 9H-carbazole with N-bromosuccinimide (NBC) [24] or its Tucker iodination reaction [25] correspondingly yields 3,6-dibromocarbazole or 3,6-diiodocarbazole. On the other hand, 2,7-dibromocarbazole is obtained by a two-step synthesis, as shown in the Scheme 1 [26]. The starting material 4,4′-dibromo-1,1′-biphenyl is firstly nitrified. The obtained 4,4′-dibromo-2-nitro-1,1′-biphenyl is then reacted with triethylphosphate to get 2,7-dibromo-9H-carbazole. The nitrogen atom of the halogenated carbazoles can be then functionalized by different alkyl or aryl groups in order to obtain the key starting materials: 3,6-diiodo-9-alkylcarbazoles (**36DICr**), 3,6-diiodo-9-arylcarbazoles (**36DI9ArCr**), 3,6-dibromo-9-alkylcarbazoles (**36DBrCr**), 3,6-dibromo-9-arylcarbazoles (**36DBr9ArCr**), 2,7-dibromo-9-alkylcarbazoles (**27DBrCr**), and 2,7-dibromo-9-arylcarbazoles (**27DBr9ArCr**), which are shown in the Scheme 1. The alkylation reactions with bromo or iodo alkanes under basic conditions are rather simple and widely described in the literature [27,28,29]. The di-halogenated 9-arylcarbazoles are prepared by Ullmann or Buchwald–Hartwig reactions [30,31,32].

**Scheme 2:**
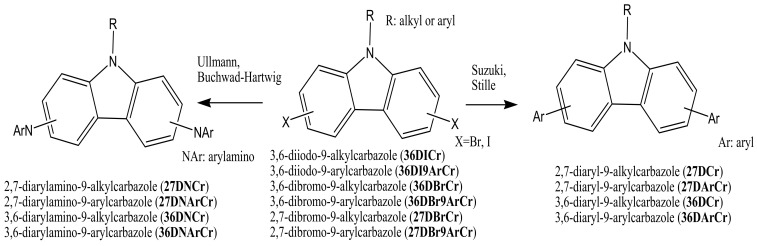
Synthetic pathway of the 2,7(3,6)-diaryl(arylamino)-substituted objective 9-alkyl(aryl)carbazoles.

The key starting di-halogenated compounds **36DICr**, **36DI9ArCr**, **36DBrCr**, **36DBr9ArCr**, **27DBrCr**, and **27DBr9ArCr** can then be used in the following amination or C-C coupling reactions to obtain the target compounds: 2,7-diarylamino-9-alkyl(aryl)carbazoles (**27DNCr** and **27DNArCr**), 3,6-diarylamino-9-alkyl(aryl)carbazoles (**36DNCr** and **36DNArCr**), 2,7-diaryl-9-alkyl(aryl)carbazoles (**27DCr** and **27DArCr**), or 3,6-diaryl-9-alkyl(aryl)carbazoles (**36DCr** and **36DArCr**) (Scheme 2). Most of the objective carbazole-based derivatives, which are used later as electroactive components for the production of OLED devices, are obtained from the di-halogenated carbazoles by the Ullmann, Suzuki, Stille, or Buchwald–Hartwig reactions.

The Ullmann reaction is a coupling reaction between aryl halides and aromatic amines or aromatic heterocyclic compounds in the presence of copper-based catalysts. The mechanism of the Ullmann reaction was extensively studied for many years. Complications arise because the reactions are often heterogeneous, especially those catalyzed with metallic copper. [33,34]. Suzuki cross-coupling procedures, developed by Nobel laureate Akira Suzuki, are among the most widely investigated reactions in the formation of carbon–carbon bonds in aromatic compounds. These reactions were generally catalyzed by inorganic catalysts, such as soluble palladium (Pd) complexes having various ligands, and more recently, also in aqueous media [35,36]. The Buchwald–Hartwig amination procedure is a chemical reaction used in organic chemistry for the formation of carbon–nitrogen bonds via palladium-catalyzed coupling reactions of aromatic amines with aryl halides [37]. Other reactions as the Stille coupling [38,39], Diels Alder [40,41,42], and Friedel–Craft [43] have also been used to obtain the target carbazole compounds, but in rarer cases. In the general case, diarylamino carbazoles (**27DNCr**, **27DNArCr**, **36DNCr**, and **36DNArCr**) are usually obtained by Ullmann or Buchwald–Hartwig reactions. On the other hand, diaryl carbazoles (**27DCr**, **27DArCr, 36DCr**, and **36DArCr**) are usually prepared by Suzuki or Stille reactions, as presented in Scheme 2.

## 3. Diaryl(diarylamino)-substituted Carbazoles as Charge-Transporting Layer Materials for OLEDs

**Scheme 3:**
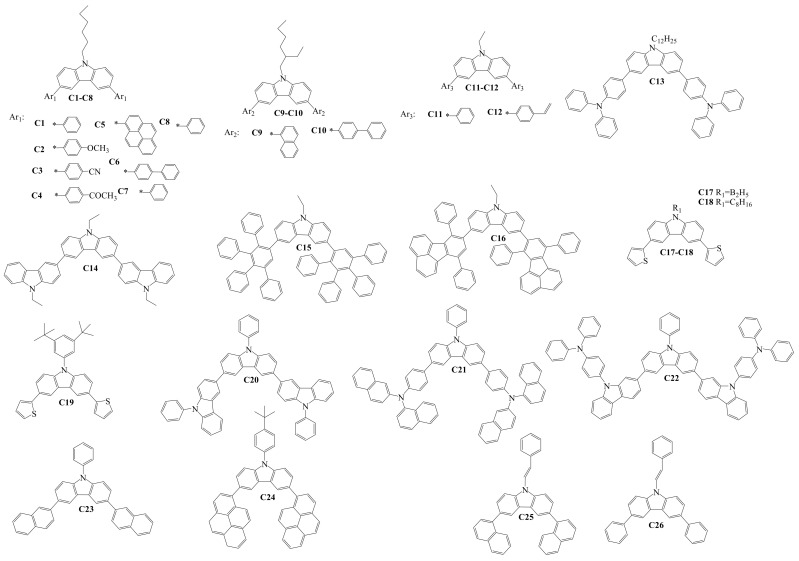
Structures of 3,6-diarylcarbazoles used for hole-transporting films in OLEDs.

Structures of the diaryl-substituted compounds (**Cx**), which were used to form the hole-transporting layers (HTLs) in OLED devices, are shown in Scheme 3. The target compounds **C1**–**C14** [44,45,46,47,48] and **C17**–**C26** [49,50,51,52,53,54] were obtained under the conditions of the Suzuki reaction. Compounds **C15**–**C16** were generated by the Diels–Alder reaction between conjugated diene and substituted alkene, forming the substituted cyclohexene fragments [55]. Authors of the research studied properties of the derivatives and used these materials for the formation of HTLs in OLED devices. Thermal properties were examined for compounds **C1**–**C13**, **C17**–**C19**, **C21**–**C22**, and **C24**–**C26**. The reported material **C22** demonstrated the highest thermal stability in this group, with a very high thermal decomposition temperature (T_d_) of 575 °C, as well as the highest glass transition temperature (T_g_) of 260 °C. The values of ionization potentials (I_p_) for compounds **C9**–**C11, C17**–**C20**, and **C23** were 5.65 eV, 5.55 eV, 5.8 eV, 5.38 eV, 5.42 eV, 5.19 eV, and 5.50 eV, respectively, and confirmed suitable hole-injecting properties for thin layers of many of the materials. HOMO and LUMO levels of the materials **C1**–**C8**, **C13**–**C17, C19**–**C22**, and **C24**–**C26** are different and depend on the nature of the substituents. The HOMO level of the diarylcarbazoles varied between −4.93 and −6.02 eV, and the LUMO level was in a broad range between −0.87 and −2.93 eV due to different electron-withdrawing or donating substituents at the carbazole core.

Positive charge drift mobility (µ_h_) in the thin layers of the derivatives **C12**, **C17**–**C19, C21**–**C22**, and **C25**–**C26** were reported. The compounds demonstrated rather high hole-drift mobility in their amorphous films ranging from 5 × 10^−5^ cm^2^·V^−1^·s^−1^ to 1.5 × 10^−4^ cm^2^·V^−1^·s^−1^ at high electrical fields. The charge-injecting/transporting properties of these materials confirmed that they are suitable hole-transporting layer materials in OLEDs.

Agarwal fabricated the single-layer spin-coated OLEDs, in which the derivatives **C1-C8** were sandwiched between indium tin oxide (ITO) or ITO/PEDOT:PSS {poly(3,4-ethylene-dioxythiophene): poly(styrenesulfonate)} as the anode and a calcium (Ca) cathode, which was protected by a thick layer of Al. Multilayer OLEDs with the materials **C1**–**C8** were also fabricated. The structure of the devices was ITO/F_4_TCNQ (2,3,5,6-tetrafluoro-7,7′,8,8′-tetracyano-p-quinodimethane)/TPD (N,N′-(bis(3-methylphenyl)-1,1′-biphenyl-4,4’-diamine))/CBP (N,N′-dicarbazolyl-4,4′-biphenyl)/**C3** or **C4**/BCP (4,7-diphenyl-1,10-phenanthroline)/LiF/Al. Brightness as high as 900–1000 cd/m^2^ was achieved in the multilayer OLED devices using the HTLs of **C3** or **C4**. Green electro-phosphorescent devices ITO/PEDOT:PSS/**C9** or **C11**/CBP/Ir(ppy)_3_ (tris(2-phenylpyrydine(iridium(III))/TPBi (2,2′,2″-(1,3,5-benzinetriyl)-tris(1-phenyl-1-H-benzimidazole))/LiF/Al were also presented. The device with **C11** demonstrated the best results, with a turn-on voltage of 5 V, a maximum brightness of 9800 cd/m^2^, and a maximum current efficiency of 22.5 cd/A. **C12** was tested as an electron-confining HTL for phosphorescent OLEDs with a red, green, or blue emitting layer. The power efficiency of the studied red device was increased from 8.5 to 13.5 lm/W, an increment of 59%, and the maximum luminance was enhanced from 13,000 to 19,000 cd/m^2^, an increment of 46%, as compared with an analogous device using commercial HTL material. For the blue device using **C12**, the power efficiency was increased from 6.9 to 8.9 lm/W, an increment of 29%, and the maximum brightness was enhanced from 9000 to 11,000 cd m^−2^, an increment of 22%. **C13** as an HTL was investigated by Kochapradist et al. Tris(8-hydroxyquinolinato)aluminum (Alq_3_) emitter-based green OLEDs, with the structure of ITO/PEDOT:PSS/**C13**/Alq_3_/LiF:Al, were fabricated. The material **C13** showed excellent hole-transporting properties for the green device—a luminance (current) efficiency exceeding 5 cd/A was achieved. Lee and co-workers studied the hole-transporting properties of **C14** and formed the device with ITO /CuPc (copper(II)phthalocyanine)/**C14**/Alq_3_/LiF/Al. For an objective comparison of electroluminescent properties, a reference device was fabricated and NPB was used as the HTL material. The device with the compound **C14** showed a high power efficiency of 1.67 lm/W and a current efficiency of 6.12 cd/A, which were higher as compared with the NPB-based device. OLEDs with the structures ITO/2-TNATA (4,4′,4′′-tris{N-(2-naphthyl)-N-phenylamino}-triphenylamine)/**C15** or **C16**/Alq_3_/LiF/Al were fabricated by Park and co-workers. The devices showed pure performance, with their luminescence efficiency exceeding only 3 cd/A and a power efficiency exceeding 1 lm/W. 

In order to identify the charge-transporting properties of **C17**, multilayer OLED devices were fabricated by Kim et al. The green device ITO/CuPc/**C17**/Alq_3_/LiF /Al showed a current efficiency of 5.16 cd/A and a power efficiency of 2.35 lm/W. Braveenth and co-authors studied the properties of the compounds **C18**–**C19** as hole-transporting materials. Device configuration was ITO/DNTPD (4,4′-bis[N-[4-{N,N-bis(3-meth ]-N-phenylamino]biphenyl)/**C18** or **C19** /Bebq_2_ (bis(10-hydroxybenzo[h]quinolinato)beryllium): Ir(mphmq)_2_(tmd) (bis[2,4-dimethyl-6-(4-methyl-2-quinolinyl)phenyl](2,2,6,6-tetramethyl-3,5-heptanedionate)/Bebq_2_/LiF/Al. A **C18**-based phosphorescent device exhibited higher maximum current efficiency (24.6 cd/A) and higher maximum external quantum efficiency (23.2%) as compared with a **C18**-based OLED. In the double-layer device, ITO/**C20**/Alq_3_/LiF/Al, in which **C20** was used as the hole-transporting material, a yellowish-green color arising from the Alq_3_ was observed. It was only reported that the luminance at the applied voltage of 10 V was about 9600 cd/m^2^. Devices with the structures of ITO/**C21**/TPBi/LiF/Al and ITO/NPB/**C21/**TPBi /LiF/Al were fabricated to evaluate the properties of **C21** as a hole-transporting light-emitting layer. A quantum efficiency of 2.21% was obtained for the **C21-**based device having an additional NPB layer. 

Kumar and co-workers investigated the hole-transporting properties of **C25** and **C26** for fluorescent OLEDs ITO/PEDOT:PSS/NPB/**C25** or **C26**/Alq_3_/LiF/Al, as well as for phosphorescent devices ITO/PEDOT:PSS/NPB/**C25** or **C26** /Ir(ppy)_3_/CBP/TPBi/LiF/Al. At 1000 cd/m^2^, the fluorescent green device with **C26** showed a current efficiency of 4.0 cd/A, which was about 135% higher than that of the typical HTM NPB-based device. Green phosphorescent devices with **C25** showed a high current efficiency of 58.4 cd/A (power efficiency 54.8 lm/W and EQE 16.1%), while 45.1 cd/A (power efficiency 40.8 lm/W and EQE 12.5%) was measured in the **C26**-containing device. 

**Scheme 4:**
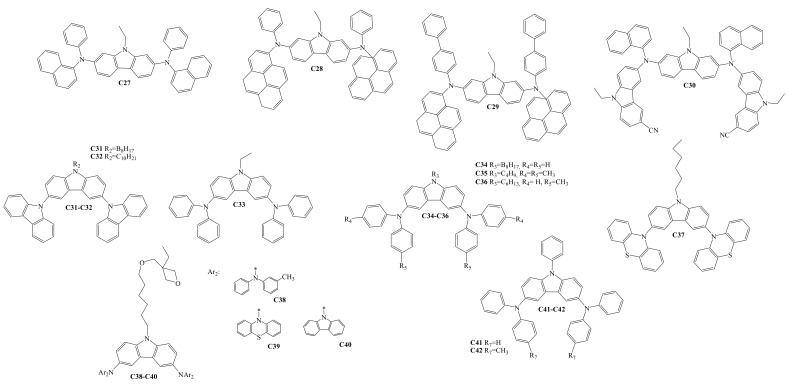
Structures of diarylamino-substituted carbazoles used for HTL formation.

Diarylamino-substituted carbazoles, which were used as HTLs in OLEDs, are shown in Scheme 4. The target compounds **C27**–**C42** were all obtained by Ullmann amination reactions [56,57,58,59,60,61,62]. **C30**–**C42** demonstrated sufficient and high thermal stability, with the thermal degradation temperatures (T_d_) ranging from 263 °C to 490 °C. The derivatives **C30**–**C42** are also suitable for glass formation, having a T_g_ in the range of 42–217 °C. The Ip of the compounds **C30**–**C31** and **C37**–**C42** were reported and demonstrated the values of 5.80 eV, 5.81 eV, 5.50 eV, 5.24 eV, 5.43 eV, 5.67 eV, 5.28 eV, and 5.34 eV, respectively. HOMO and LUMO energy levels were reported for the materials **C27**–**C30**. The values of HOMO/LUMO for the derivatives **C27**–**C30** were −4.92/−1.91 eV, −4.92/−2.24 eV, −4.92/−2.33 eV, and −4.83/−1.87 eV, respectively.

The charge-transporting properties of the derivatives **C27**–**C28** and **C31**–**C42** were studied with the time-of-flight technique. The µ_h_ values ranged from 1.2 × 10^−8^ to 2 × 10^−3^ cm^2^·V^−1^·s^−1^ at high electric fields. The µ_h_ in the films of **C32** and **C40** dispersed in bisphenol Z polycarbonate (PC-Z) reached 10^−3^ cm^2^·V^−1^·s^−1^ at high electrical fields. The charge injection/transport characteristics of some from the derivatives **C27**–**C28** and **C31**–**C42** showed that they are suitable as hole-transporting layers for OLED devices.

Shen and co-authors investigated two types of devices. The structure of device I was ITO/**C27**–**C30**/TPBi/Mg:Ag, and that of device II was ITO/**C27**–**C30**/(Alq_3_)/Mg:Ag. In type I, the derivatives functioned as both hole-transporting and emitting materials. In the type II devices, light was emitted from either the disubstituted carbazole layers or from Alq_3_. Device II with **C30** reached a maximum external quantum efficiency of 1.3%.

## 4. Diaryl(arylamino)-substituted Carbazoles as Host Materials for PhOLEDs

**Scheme 5:**
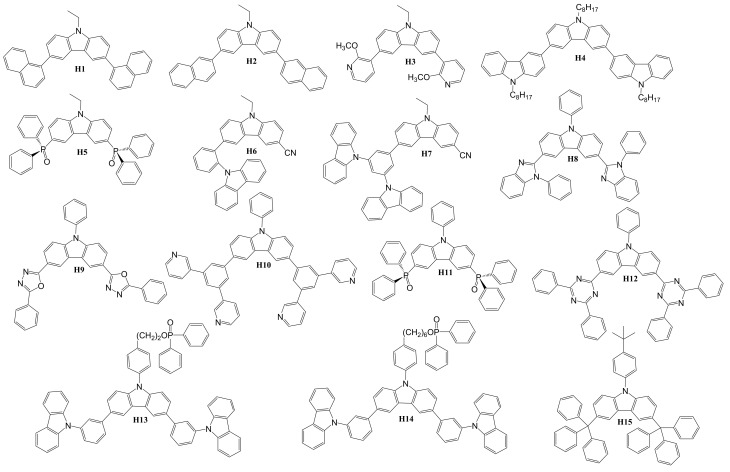
Chemical structures of diarylcarbazoles used as host materials for PhOLEDs.

The structures of the diaryl-substituted carbazoles, which were used as host materials for PhOLED devices, are shown in Scheme 5. The compounds **36DICr**, **36DBrCr**, and **36DBr9ArCr** were firstly obtained for their synthesis as key starting materials, as shown in Scheme 1. The objective compounds **H1**–**H4** [63,64,65], **H6**–**H7** [66], **H10** [67], and **H12**–**H14** [68,69] were obtained during the Suzuki reaction. The diphosphine oxide-based material **H5** was obtained by the oxidation of the corresponding diphosphine, which was prepared by a lithium–halogen exchange reaction between the di-brominated aromatic bridge and n-butyl lithium, followed by a reaction with chlorodiphenylphosphine [70]. Compound **H8** was obtained when 9-phenyl-9-carbazole-3,6-dicarboxylic acid reacted with SOCl_2_, and then the resulting compound was treated with trimethylamine and 2-aminodiphenylamine. Derivative **H9** resulted from the reaction of 9-phenyl-3,6-di(2H-tetrazol-5-yl)-9H-carbazole with the o-xylene solution containing benzoyl chloride [71]. **H11** was synthesized by the coupling reaction of chlorodiphenylphosphine with 3,6-dibromo-N-phenylcarbazole [72]. The reaction of 9-(4-tert-butylphenyl)carbazole with triphenylmethanol in the presence of Eaton’s reagent yielded the objective derivative **H15** [73].

The destruction temperatures (T_d_) of the compounds **H1**–**H4, H6**–**H10**, and **H12**–**H15** were reported. The compound **H7** demonstrated a rather low thermal destruction temperature of 186 °C, while the temperatures of the other compounds reached 300 °C. All the compounds **H1**–**H15** were obtained as amorphous substances and could form amorphous glasses with T_g_ values ranging from 30 °C to 269 °C. The HOMO and LUMO energy levels of **H1**–**H15** were reported. The HOMO/LUMO energies of the derivatives **H1**–**H15** were different and depended on substituents at the 3,6 positions of the carbazole core. The HOMO level was between −5.21 and −6.48 eV, and the LUMO level varied between −1.90 and −2.83 eV. The values of µ_h_ in the thin films of the prepared materials **H3** and **H15** were investigated. The amorphous layers of these materials showed very similar results, and the charge drift mobility reached 5 × 10^−4^ cm^2^·V^−1^·s^−1^ at high electrical fields.

Kim et al. investigated the phosphorescent devices ITO/NPD/**H1** or **H2**: Ir(ppy)_3_/Alq_3_/LiF/Al. A maximum brightness of 4165 cd/m^2^, low current efficiency of 1.58 cd/A, and also a low external quantum efficiency of 0.59% were achieved in the group of the most efficient device with the host **H1** [74]. The PhOLEDs ITO/PEDOT/**H3**:Ir(ppy)_3_/TPBi/LiF/Al containing the green emitter Ir(ppy)_3_ in hosts **H3** using wet and dry processes as well as dry process, with the incorporation of an additional TAPC hole-transporting layer, were fabricated by Jou and co-workers. The efficiency of the best device was reported as 11.6 lm/W (21 cd/A). The host material **H4** was used in a solution processable multilayer device ITO/PEDOT/ **H4** Ir(ppy)_3_/TPBi /LiF/Al. Its maximum photometric efficiency was 35.5 cd/A, and the maximum EQE exceeded 10.6%. Sapochak et al. investigated the blue devices ITO/TCTA/**H5:** FIrpic/DPDT (2,8-bis(diphenylphosphoryl)dibenzo[b,d]thiophene)/LiF/Al and obtained an EQE of 7% at a voltage of about 7 V [75]. The simple-structured blue PhOLEDs with hosts **H6** and **H7** and FIrpic dopant correspondingly reached a maximum efficiency of 43.9 cd/A and 46.1 cd/A. Sky blue PhOLEDs were fabricated using either FIrpic or (DFPPM)_2_Irpic as phosphorescent emitters co-evaporated with the hosts **H8** or **H9** in the devices. The host material **H9** gave excellent OLED characteristics, with an EQE of 17.7% for green and 20.6% for the red device. Jiang et al. investigated the host **H10** and reported that the device with FIrpic as a dopant reached a maximum current efficiency of 7.1 cd/A. Ahn and co-workers formed the device ITO/HATCN (1,4,5,8,9,11-hexaazatriphenylenehexacarbonitrile)/TAPC/(3,5-di(carbazol-9-yl)-N,N-diphenylaniline) DCDPA/**H11**:TSPO1 (diphenyl-4-triphenylsilylphenyl-phosphine oxide)/TPBi/LiF/Al and found that the multilayer PhOLED shows a very high external quantum efficiency of 27.8% [76]. A green PhOLED with the structure ITO/2-TNATA/NPB/**H12**/TPBi/LiF/Al was fabricated by Song et al. The device showed that its maximum luminescence efficiency could exceed 29 cd/A and its EQE could reach 8.68%. The double-layer solution-processed blue PhOLEDs ITO/PEDOT:PSS/PVK/**H13** or **H14**: FIrpic/Ba/Al were fabricated. The best results yielded a device with an **H14** host-maximal current efficiency of 4.16 cd/A. Tsai et al. created the device ITO/PEDT:PSS/DPAS (2,2′-bis(N,N-diphenylamine)-9,9′-spirobifluorene)/TCTA/**H15**:FIrpic/TAZ (3-(4-biphenylyl)-4-phenyl-5-(4-tert-butylphenyl)-1,2,4-triazole) /LiF /Al. The efficiency of the **H15**-based device reached 7% at 100 cd/m^2^.

**Scheme 6:**
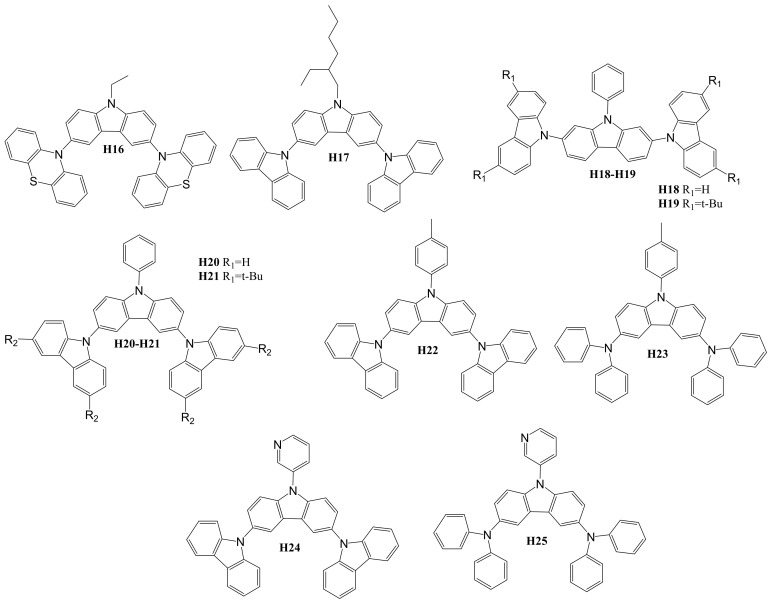
Structures of diarylamino-substituted carbazoles used as host materials.

The structures of the diarylamino-substituted carbazoles, which were used as host materials for PhOLED devices, are shown in Scheme 6. The objective compounds **H16**–**H17** [77,78], **H22**–**H23** [79], and **H24** [80] were obtained by Ullmann reaction. The compounds **H18**–**H21** [81] and **H25** [57] were prepared by the Buchwald–Hartwig amination procedure. TGA confirmed that the compounds are highly and thermally stable. The T_d_ values of the derivatives **H16**–**H23** were 419 °C, 384 °C, 364 °C, 389 °C , 399 °C, 417 °C, 335 °C, and 404 °C, respectively. The T_g_ values of the materials **H16**–**H23** ranged from 81 °C to 210 °C. I_p_ was reported for some of the derivatives. For example, the phenothiazine-substituted compound **H16** had a lower I_p_ of 5.25 eV as compared with the carbazole-based compound **H17** (5.57 eV). The described HOMO and LUMO energies of the compounds **H17** and **H19**–**H25** had different values and depended considerably on arylamino fragments. The energy levels of these derivatives ranged from −4.86 to −5.62 eV for HOMO and from −1.78 to −2.74 eV for LUMO.

Bagdziunas et al. fabricated three PhOLEDs in which **H16** was used as host material. The structures of the devices were ITO/**H16**:Ir(ppy)_3_/Bphen (4,7-diphenyl-1,10-phenanthroline)/Ca:Al or ITO/m-MTDATA/**H16** :Ir(ppy)_3_/Bphen/Ca:Al or ITO/m-MTDATA/**H16** :Ir(piq)_2_(acac)/Bphen/Ca:Al. The maximum power and EQE up to 47.5/29.6 lm/W and 20.0/10.5% for green or red PhOLED devices, respectively, were reported in the most effective device. The structure ITO/PEDOT: PSS/**H17**: Ir(Fppy)_3_/LiF/Al was fabricated by Pudzs et al. [82]; however, the un-optimized OLED reached very low efficiencies of only 0.07 lm/W and 0.45 cd/A. The reported optimized phosphorescent device with the host **H20** material ITO/PEDOT:PSS/TAPC/ **H20** :FIrpic/TmPyPB/LiF/Al [83] had a maximum current efficiency of 36 cd/A at 200 cd/m^2^. A device stack with the carbazole-based host **H22** doped with an orange phosphor tris(2-phenylquinoline)iridium(III) [Ir(2-phq)_3_] demonstrated improved efficiency (7.4% and 16 lm/W). Most importantly, the superior stability of the device using **H22** enabled a lifetime well above 10,000 h at a practical luminance of 1000 cd/m^2^ [84]. PhOLED devices ITO/PEDOT/PSS/TAPC/**H24** or **H25**: Ir(ppy)_3_ /BmPyPb/LiF/Al were fabricated by Park and Lee. The green device with **H24** showed the best performance, with a maximal EQE of 21.3%.

## 5. Diaryl(arylamino)-substituted Carbazoles as Fluorescent Emitters of OLEDs

**Scheme 7:**
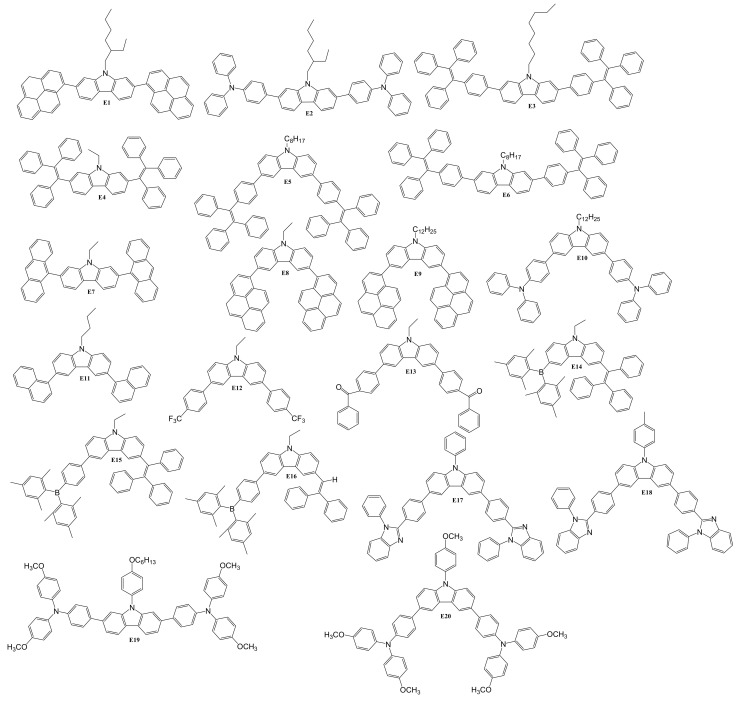
Structures of 2,7(3,6)-diarylcarbazole-based emitters.

Structures of the 2,7-diaryl- or 3,6-diaryl-substituted carbazoles, which were used as emitters (**E**) in the fluorescent OLED devices, are shown in Scheme 7. All the target diaryl-substituted compounds **E1**–**E13** [85,86,87,88,89,90,91,92,93] and **E15**–**E20** [94,95,96,97], except for **E14**, were obtained during the Suzuki reaction of the di-halogenated carbazoles with the corresponding boronic acids or their esters. The objective material **E14** was synthesized by fusing triphenyl-ethylene and one dimesitylboron group moiety into a system, with one attached to the carbazole fragment [98].

The thermal properties of some of the emitters were investigated [99]. It was described that the compounds **E1**–**E7**, **E9**–**E10**, **E14**, and **E17**–**E18** exhibited rather high thermal stability, with a T_d_ value ranging from 283 to 603 °C. Meanwhile, the T_d_ values of the compounds **E15** and **E16** were lower and reached 219 °C and 188 °C, respectively. It was mentioned that the combination of fragments of carbazole with various conjugated aromatic fragments is favorable for the formation of homogeneous thin films with high T_g_ temperature values. The T_g_ of **E1**, **E3**–**E6**, **E9**–**E10**, **E14, E17**, and **E18** were 105, 138, 65, 138, 77, 70, 211, 88, 170, and 172 °C, respectively. The values of I_p_ for the derivatives **E1**–**E2** and **E19**–**E20** were 5.70 eV, 5.56 eV, 5.17 eV, and 5.30 eV, respectively. It was evident that the attached fragments at the 2,7- and 3,6-positions of the carbazole core provided an effective tool for the HOMO and LUMO of the compounds. The HOMO and LUMO of **E2**–**E7** and **E9**–**E20** ranged from −4.43 to −5.66 and from −2.61 to −3.01 eV, respectively. Hole drift mobilities in the amorphous layer of the compounds **E17**–**E18** were reported, and the values varied from 1 × 10^−5^ to 3 × 10^−8^ cm^2^·V^−1^·s^−1^ at a high electric field.

The device ITO/PEDOT:PSS/CBP: **E2**/TPBi/LiF/Al was fabricated by Konidena and co-workers. This OLED reached a power efficiency of only 0.5 lm/W and a current efficiency of 1.1 cd/A. The same structure device just using the emitter **E5** reached a power efficiency of 3.9 lm/W and a current efficiency of 4.9 cd/A, while the same device with **E7** reached a power efficiency of 4.12 lm/W and a current efficiency of 9.52 cd/A. Dang and co-workers experimented with the device ITO/HATCN (dipyrazino[2,3-f:2′,3′-h]quinoxaline-2,3,6,7,10,11hexacarbonitrile)/NPB/TCTA/**E3** or **E4**/TPBi/LiF/Al. The material **E4**, with a much balanced molecular conjugation and a twisted molecular conformation, exhibited much better performance in non-doped OLEDs with maximum photometric efficiency, power efficiency, and EQE values of 7.38 cd/A, 6.81 lm/W, and 3.0%, respectively. The simple-structured ITO/**E8**/TPBi/Mg:Al demonstrated a power efficiency of 2.55 lm/W and a current efficiency of 4.75 cd/A. OLEDs with the structure ITO/PEDOT:PSS/**E9** or **E10**/NPB/CPB/LiF/Al were characterized by a power efficiency of 1.68 or 2.67 lm/W and a current efficiency of 3.55 or 5.89 cd/A, respectively. Feng et al. studied the properties of the device ITO/MoO_3_/TAPC/TCTA/**E11**/TPBi/LiF/Al. The designed emitter showed a bright violet light at a wavelength of 400 nm, with a current efficiency of 0.65 cd/A and an EQE of about 2%. To examine the electroluminescent properties of the emitters **E12** and **E13**, researchers fabricated a device by solution processing with the following configuration: ITO/PEDOT:PSS/ NPB/**E12** or **E13**/BCP/Alq_3_/LiF/Al. The white light emission from the device was stable in the range from 5 V up to 20 V. The maximal luminance values were found to be 20 cd/m^2^ and 110 cd/m^2^, respectively, for the **E12** and **E13-**based devices. Multilayer, non-doped OLEDs, with a configuration of ITO/HATCN/ NPB/**E14**, **E15** or **E16**/TPBi/LiF/Al, were fabricated. They exhibited good performance with low turn-on voltages of 4.2 V, 6.2 V, and 6.0 V, high maximal luminance values of 13,930 cd/m^2^, 21,054 cd/m^2^, and 4376 cd/m^2^, and maximum current efficiency values of 4.74 cd/A, 3.34 cd/A, and 2.34 cd/A, respectively. A three-layer PhOLED ITO/PEDOT:PSS/**E17**:(pbi)_2_Ir(acac)/TPBi/LiF/Al was fabricated by Hung and co-authors. The maximum value for energetic efficiency of 62 lm/W and 62 cd/A for current efficiency were achieved from the system at a brightness of 120 cd/m^2^. The structure PEDOT:PSS/TCTA/**E18** Ir(ppy)_3_:PQ2Ir(acac)/TPBI/CsF/Al was also fabricated. Hybrid white emitting devices with **E18** showed the peak external quantum efficiency exceeding 10% and a power efficiency of 14.8 lm/W, at a luminance of 500 cd/m^2^. Skorka et al. investigated two devices: ITO/PEDOT:PSS/PVK: PBD: **E19**/Ca/Ag and ITO/PEDOT:PSS/PVK: PBD **E19**/LiF/Al. The current efficiency measured at ca. 9 V was about 0.55 cd/A for the device with the Ca/Ag cathode, and about 0.70 cd/A for that with the LiF/Al cathode.

**Scheme 8:**
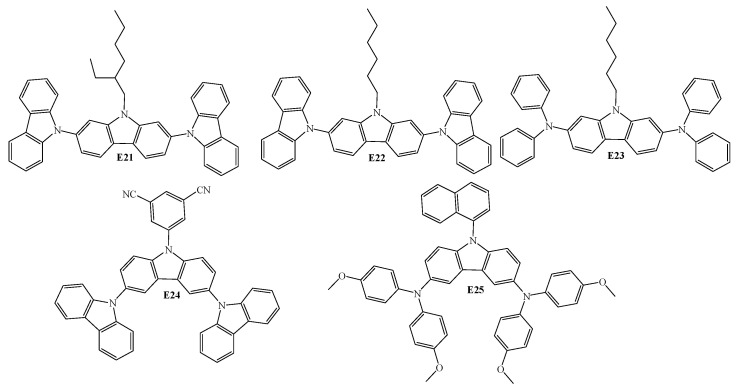
Structure of diarylamino-substituted carbazoles as fluorescent emitters for OLED devices.

The 2,7-di(arylamino)- or 3,6-di(arylamino) carbazoles, which were used as fluorescent emitters (**Ex**) in the OLED devices, are shown in Scheme 8. The compounds **27DBrCr**, **36DBr9ArCr**, or **36DI9ArCr** were firstly obtained as starting materials for the synthesis, as shown in Scheme 1. The target compounds **E21** [100,101] and **E23** [102] were obtained during the Buchwald–Hartwig amination reaction, and the materials **E22** [79], **E24** [103], and **E25** [104] were obtained during the Ullmann reaction of the starting derivatives.

It was reported that the compounds **E21**–**E25** have high thermal stability, with temperatures of decomposition at 436, 440, 387, 285, and 413 °C, respectively. The derivatives **E21**, **E24**, and **E25** are also suitable for glass formation, with T_g_ values at 88, 110, and 119 °C. HOMO and LUMO energy levels were described for all the molecules of **E21**–**E25**. The HOMO/LUMO values of the molecules were −5.49/−2.13 eV, −5.29/−1.89 eV, −5.25/−2.21 eV, −5.57/−3.26 eV, and −4.94/−2.13 eV, respectively. The electric field dependencies on positive charge drift mobility were characterized in the layers of the material **E21**. The derivative **E21** containing two carbazolyl fragments connected to a central carbazole core demonstrated the hole mobility of 2 × 10^−3^ cm^2^·V^−1^·s^−1^ at a high electric field.

Luka et al. investigated the properties of a UV OLED structure containing an indium-free transparent anode (ZnMgO:Al) and 2,7-di(9-carbazolyl)-9-(2-ethylhexyl)carbazole **E21** as a light-emitting layer. The obtained device was characterized by a high turn-on voltage of ca. 9 V. The driving voltage required for the device to operate at a current of 20 mA/cm^2^ was 10.5 V [105]. Li and co-authors studied the properties of **E22** and **E23**, and suggested that the new compounds have potential applications as blue light emitters in the field of OLEDs. With the emitter **E24**, the device ITO/HAT-CN/NPB/TAPC/PPT:**E24**/PPT/TPBi/LiF/Al was described. The OLED displayed excellent device performance, with a power current efficiency of 15.26 cd/A, a power efficiency of 8.14 lm/W, a maximum external quantum efficiency of 13.33%, and a maximal brightness of 1526 cd/m^2^. A single-layer OLED with the structure ITO/CuI/**E25**/Ca:Al and the bilayer device ITO/CuI/**E25**/Bphen/Ca:Al were investigated by Deksnys and co-authors. The bilayer structure had a high photometric efficiency of 10.5 cd/A, a maximum luminance of 27,000 cd/m^2^ (at 15 V), and an EQE of ca. 3.3%.

## 6. Diary(arylamino)-substituted Carbazoles as TADF Emitters for OLEDs

**Scheme 9:**
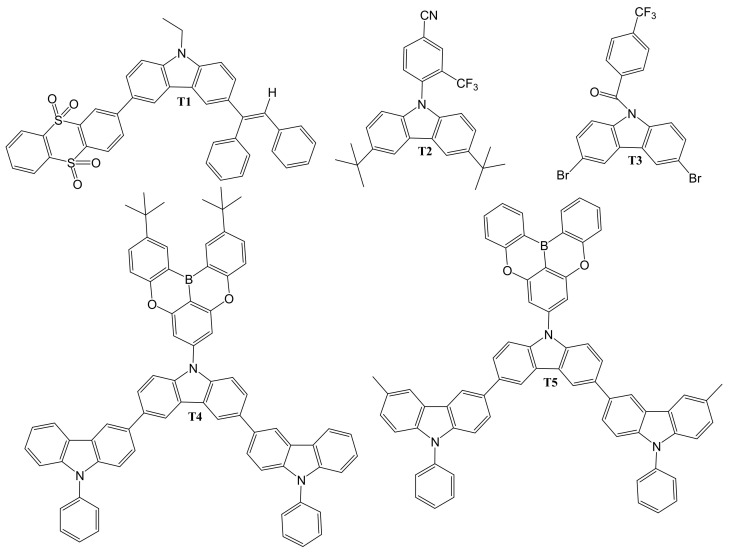
Structure of 3,6-diarylcarbazoles as TADF emitters for OLED devices.

It should be mentioned that only 3,6-diaryl-substituted carbazoles are still reported as the TADF compounds suitable for emitting layers (**Tx** in Scheme 9). The starting materials (**36DBrCr** and **36DBr9ArCr**) for the synthesis of the emitters as objective derivatives were prepared, as shown in Scheme 1. The target compound **T1** was obtained during the Suzuki reaction [106]. The compounds **T2**–**T3** were obtained by nucleophilic substitution [107,108]. Other compounds, **T4**–**T5**, were obtained during the Buchwald–Hartwig reaction [109].

The T_d_ of compounds **T1**–**T3** were very high and reached 178 °C, 242 °C, and 287 °C, respectively. These destruction temperatures were lower than those of compounds **T4**–**T5**, where they exceeded 500 °C. The Ip of the studied derivative **T2** in solid-state was reported as 5.89 eV. The values of HOMO/LUMO for compounds **T1**, **T4**, and **T5** were −5.77/−2.40 eV, −5.63/−2.64 eV and −5.62/−2.67 eV. Only the charge transporting properties for layers of compounds **T2** were described. They exhibited a bipolar charge transport with balanced hole and electron mobility exceeding 10^−4^ cm^2^·V^−1^·s^−1^ at higher than 3 × 10^5^ V/cm electric fields. 

The literature reported the TADF-based device ITO/HATCN/NPB/ **T1**/TPBi/LiF/Al, which showed green-bluish light with a 5 V turn-on voltage, a maximum luminance of 8264 cd/m^2^, and a maximum photometric efficiency of 3.96 cd/A. While the blue device ITO/MoO_3_/TCTA/mCP/**T2**/TSPO1/TPBi/Ca/Al demonstrated a turn-on voltage of 4.4 V, a maximum brightness of 3100 cd/m^2^, and a photometric efficiency of 5.4 cd/A. Multilayer blue TADF OLEDs with a configuration of ITO/HATCN/TAPC/DCDPA/DBFPO:**T4** or **T5**/DBFPO /TPBi/LiF/Al were fabricated by Karthik et al. These blue devices showed maximal EQE/photometric efficiency/luminance values of 30.7%/46.7cd/A/18,160 cd/m^2^, and 29.1%/36.4 cd/A/11,690 cd/m^2^ for the **T4** and **T5-**based emitters, respectively.

**Scheme 10:**
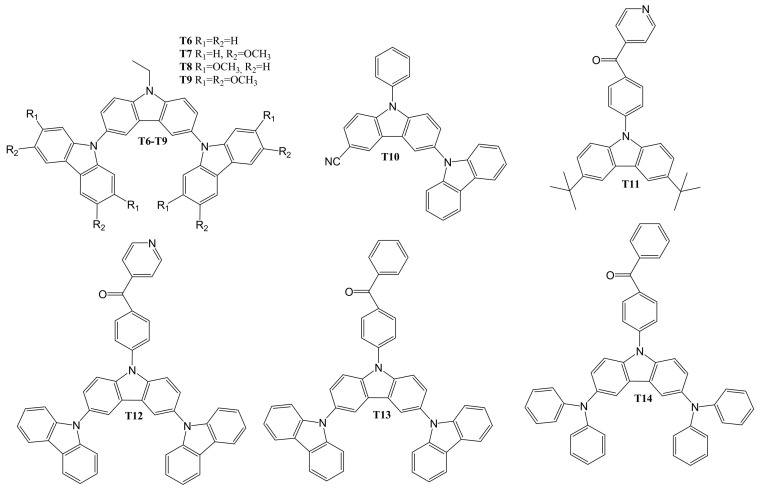
Structures of 3,6-di(arylamino)-substituted carbazoles as TADF emitters for OLED devices.

The 3,6-Di(arylamino)carbazoles, which were used as the TADF emitters for OLEDs, are shown in Scheme 10. The target compounds **T6**–**T12** were obtained during the Ullmann amination reaction of the corresponding diiodo or dibromo derivatives shown in Scheme 1 [110,111,112]. The compounds **T13**–**T14** were synthesized during the Buchwald–Hartwig reactions of the starting materials [113].

Thermal stability was presented for the conjugates **T6**–**T9** and **T11**–**T14**. These derivatives have rather high stability, with T_d_ values from 291 °C to 530 °C. The highest T_d_ value, which exceeded 530 °C, was mentioned for derivative **T12**. DSC measurements showed that the conjugates **T11**–**T14** formed amorphous films, with the T_g_ ranging from 86 °C to 155 °C. The derivatives **T13**–**T14** containing carbazolyl or diphenylamino fragments had the highest T_g_ values, reaching 155 °C. The HOMO and LUMO energies of **T11**–**T14** were presented to be −5.60/−2.63 eV, −5.53/−2.58, −5.06/−2.31 eV, and −5.17/−2.24 eV, respectively. 

In order to evaluate the potential of the mentioned TADF emitters for OLEDs, authors fabricated devices by using the synthesized TADF materials. Compounds **T6**–**T9** were tested in TADF-based devices of the following structures: ITO/MoO_3_/one of **T6**–**T9**/TCz1:FIrpic/TSPO1/TPBi/Ca:Al. The most promising OLED reached a current efficiency of 46.3 cd/A, a power efficiency of 33.2 lm/W, a maximum external quantum efficiency of 20.5%, and a brightness exceeding 3300 cd/m^2^. Devices with **T11**–**T12** were fabricated by using the following configuration: ITO/NPB/TAPC/mCBP/**T11** or **T12**/PPT/TmPyPb/LiF/Al. The OLEDs using **T11** and **T12** showed very different maximum EQEs of 9.4 and 23.9%, respectively. Meanwhile, current efficiencies and power efficiencies of the devices were 16.3 and 56.5 cd/A with **T11**, and 14.6 and 50.6 lm/W with **T12**, respectively. In order to study the light-emitting performance of compounds **T13**–**T14**, several devices were designed and fabricated by Zhao et al. with the TADF emitters. Device A was ITO/TAPC:**T14**/TmPyP/LiF/Al and device B was ITO/TAPC:**T13**/TmPyPB/LiF/Al; both were fabricated using different concentrations of doping ranging from 3 to 30 wt % of the emitters. The OLED B with 15 wt % of the dopant **T13** displayed excellent device performance, with a current efficiency of 72.1 cd/A, a power efficiency of 61.5 lm/W, a maximum external quantum efficiency of 22.5%, and a brightness exceeding 42,700 cd/m^2^.

## 7. Concluding Remarks

Recent developments on 2,7(3,6)-diaryl(arylamino)-substituted carbazole-based electroactive derivatives are presented here with a short description of their preparation, physical properties, and the characteristics of organic light emitting diodes using the materials. The reviewed derivatives had different functions in the OLED devices, including hole transport in the thin layers of the materials, electroluminescence or thermally activated delayed fluorescence in emitting layers as well as host functions for phosphorescent dopants. Some of the derivatives function very effectively as positive charge-transporting layer compounds, enhancing quantum efficiencies, lowering driving voltages, and increasing the life time of the OLEDs. Positive charge drift mobility in the thin films of the most effective derivatives can exceed 2 × 10^−3^ cm^2^·V^−1^·s^−1^ at high electrical fields.

Among various carbazole-based host derivatives, the diaryl-substituted conjugates are very effective as hosts for the blue (EQE > 27%), green (EQE > 210%), and red (EQE > 20%) phosphorescent organic light emitting diodes. As derivatives for the light emitting layer, the diaryl(arylamino)-substituted carbazoles cover the broad range of emitted lights, from the color blue to green, through the substitution and introduction of different aromatic fragments into the carbazole core at the 2,7(3,6) positions. For example, by using the thermally activated delayed fluorescence (TADF) function of the carbazole-based emitters, blue devices showed a maximum EQE efficiency that exceeded 30%.

Therefore, the low molar mass 2,7(3,6)-diaryl(arylamino)-substituted carbazoles are promising as charge-transporting layer derivatives, emitters, and hosts for various configurations of OLED devices, and further research in the field of new carbazole-based electroactive materials is actively ongoing to improve the characteristics of future OLED devices.

## Abbreviations

NBC	N-bromosuccinimide
36DICr	3,6-diiodo-9-alkylcarbazoles
36DI9ArCr	3,6-diiodo-9-arylcarbazoles
36DBrCr	3,6-dibromo-9-alkylcarbazoles
36DBr9ArCr	3,6-dibromo-9-arylcarbazoles
27DBrCr	2,7-dibromo-9-alkylcarbazoles
27DBr9ArCr	2,7-dibromo-9-arylcarbazoles
27DNCr	2,7-diarylamino-9-alkylcarbazoles
27DNArCr	2,7-diarylamino-9-arylcarbazoles
36DNCr	3,6-diarylamino-9-alkylarylcarbazoles
36DNArCr	3,6-diarylamino-9-arylcarbazoles
27DCr	2,7-diaryl-9-alkylcarbazoles
27DArCr	2,7-diaryl-9-arylcarbazoles
36DCr	3,6-diaryl-9-alkylcarbazoles
36DArCr	3,6-diaryl-9-arylcarbazoles
Cx	charge transporting compound
HTL	hole transporting layer
T_d_	thermal decomposition temperature
T_g_	glass transition temperature
I_p_	ionization potential
HOMO	the highest occupied molecular orbital
LUMO	the lowest unoccupied molecular orbital
µ_h_	hole drift mobility
ITO	indium tin oxide
PEDOT:PSS	poly(3,4-ethylene-dioxythiophene): poly(styrene-sulfonate)
F_4_TCNQ	2,3,5,6-tetrafluoro-7,7′,8,8′-tetracyano-p-quinodimethane
TPD	N,N′-bis(3-methylphenyl)-1,1′-biphenyl-4,4′-diamine
CBP	N,N′-dicarbazolyl-4,4′-biphenyl
BCP	4,7-diphenyl-1,10-phenanthroline
Ir(ppy)_3_	tris(2-phenylpyrydine(iridium(III)
TPBi	2,2′,2″-(1,3,5-benzinetriyl)-tris(1-phenyl-1-H-benzimidazole)
Alq_3_	tris(8-hydroxyquinolinato)aluminum
CuPc	copper(II)phthalocyanine
2-TNATA	4,4′,4’′-tris{N-(2-naphthyl)-N-phenylamino}-triphenylamine
DNTPD	4,4′-bis[N-[4-{N,N-bis(3-methylphenyl)amino}phenyl]-N-phenylamino]biphenyl
Bebq_2_	bis(10-hydroxybenzo[h]quinolinato)beryllium)
Ir(mphmq)_2_(tmd)	bis[2,4-dimethyl-6-(4-methyl-2-quinolinyl)phenyl](2,2,6,6-tetramethyl-3,5-heptanedionate
PC-Z	bisphenol Z polycarbonate
HATCN	1,4,5,8,9,11-hexaazatriphenylenehexacarbonitrile
DCDPA	3,5-di(carbazol-9-yl)-N,N-diphenylaniline
TSPO1	diphenyl-4-triphenylsilylphenyl-phosphine oxide
PhOLED	Phosphorescent organic light-emitting diodes
TAZ	3-(4-biphenylyl)-4-phenyl-5-(4-tert-butylphenyl)-1,2,4-triazole
TGA	thermogravimetric analysis
DSC	differential scanning calorimetry
Bphen	4,7-diphenyl-1,10-phenanthroline
Ir(2-phq)_3_	tris(2-phenylquinoline)iridium(III)
HATCN	dipyrazino[2,3-f:2′,3′-h]quinoxaline-2,3,6,7,10,11hexacarbonitrile
EQE	external quantum efficiency
TADF	thermally activated delayed fluorescence
